# Mendelian randomization study of the genetic interaction between psoriasis and celiac disease

**DOI:** 10.1038/s41598-022-25217-y

**Published:** 2022-12-13

**Authors:** Lin Li, Lixin Fu, Liwen Zhang, Yanyan Feng

**Affiliations:** Department of Dermatology, Chengdu Second People’s Hospital, Chengdu, Sichuan China

**Keywords:** Genetics, Diseases

## Abstract

Epidemiological studies have observed some relationship between psoriasis and celiac disease (CD), while the causal link between these 2 autoimmune diseases was unclear. In the current study, we aimed to explore the causal link between psoriasis and celiac disease with bidirectional 2-sample Mendelian Randomization (MR) study. Eligible instrument variables (IVs) with genome-wide significance (p < 5E−08) were extracted from the summary-level datasets from the published genome-wide association studies (GWAS), which were conducted in the European population. The inverse variance weighted (IVW) method was performed as the main analysis, sensitivity analyses and post-MR analyses were also performed. Our MR analyses found that genetically doubling the odds of CD would increase the risk for psoriasis (p = 1.58e−03, OR [95% CI] 1.232 [1.061–1.432]). And the results were supported by sensitivity analyses. While we found that genetically determined psoriasis was not associated with the risk for CD (IVW: p = 0.985, OR [95% CI] 1.000 [0.965–1.037]). Our study provided novel genetic evidence that patients with CD were at an increased risk of developing psoriasis, while psoriasis was not associated with the risk for CD. Clinicians should be aware of the associations and pay attention to skin manifestations in patients with CD.

## Introduction

Psoriasis is a chronic immune-mediated and genetic disease that affects approximately 125 million people worldwide, which mainly manifests in the skin or joints or both^1^. The pathological mechanism of psoriasis is complex and remains unclear, while dysregulation of the innate and adaptive components of the immune system in psoriasis is now widely accepted^2^. Celiac disease (CD) is also an autoimmune disease characterized by a specific serological and histological profile triggered by gluten ingestion in genetically predisposed individuals^3^.

Psoriasis and CD both are associated with multiple autoimmune disorders, and association between psoriasis and CD has been explored by several observational studies, while the results were inconsistent^4–7^. Moreover, a comprehensive meta-analysis has found that psoriasis patients were 2.16-fold more likely to be diagnosed with CD, and the CD patients were 1.8-fold more likely to have psoriasis^8^. However, limitations should be acknowledged in observational studies, for example, it was difficult to control confounding factors, such as environmental factors, education levels, socio-economic status, et al. Moreover, it was unable to fully understand the true causal effect in observational studies because there might be a reverse causal effect.

**Figure 1:**
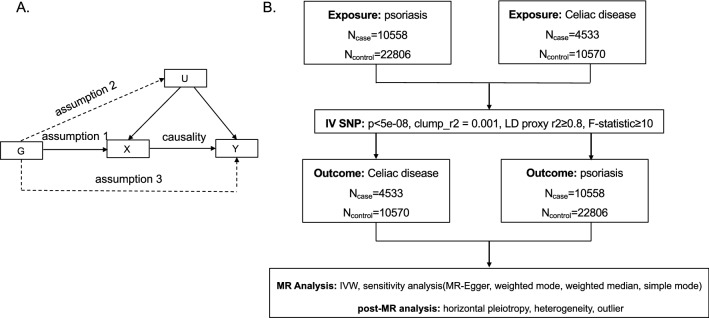
(**A**) Schematic diagram of the hypothetical relationship between genetic variant (G), exposure (X), and outcome (Y), in the presence of unobserved confounding factors (U). Solid arrows represent allowed associations between the variables, while dashed lines represent associations that are needed to be avoided for G to qualify as a robust instrumental variable (IV). (**B**) Diagram of the process for the bidirectional 2-sample MR analysis.

To overcome the limitations in observational studies, Mendelian randomization (MR) was developed. As illustrated in Fig. 1A, MR is a genetic method that applies genetic variants (G) strongly associated with the exposure (X) as instrumental variables (IVs) in a non-experimental design to assess the causal effect of exposures (X) on outcomes (Y)^9^. The most important and fundamental step of MR is selecting eligible IVs (G), which are required to meet the following 3 assumptions: assumption 1 is that IVs should be related to exposure; assumption 2 is that IVs should be unrelated to any other risk factor/confounding that may affect the outcome variable, and assumption 3 is that IVs should not have a direct association with outcome^10^. Under these circumstances, we can calculate causality between X and Y using G. Notably, the direction of G → X → Y could ensure the direction of causality and avoid reverse causation. Moreover, assumption 2 could avoid confounding factors, and the test of horizontal pleiotropy in MR can also avoid confounding factors^9^. Therefore, MR has been widely used in identifying the causal relationship between risk factors and diseases^11,12^.

Few MR studies have been performed on psoriasis, which provided novel genetic evidence of the association between psoriasis and some risk factors, including type 2 diabetes^13^, interleukin 17 level^14^ and body mass index^15^. No MR study has been performed to explore the association between psoriasis and CD. In the current study, we aimed to systematically investigate the bidirectional genetic causality between psoriasis and CD with a bidirectional 2-sample MR design.

## Material and methods

The flow chart of the study was shown in Fig. 1B.

### GWASs dataset

GWAS summary statistics were obtained from published GWASs for psoriasis^16^ (N_case_ = 10,558, N_control_ = 22,806) and CD^17^ (N_case_ = 4533, N_control_ = 10,570). Briefly, the GWAS of psoriasis was combined from three existing GWAS datasets (referred to as Kiel, the Collaborative Association Study of Psoriasis and the Wellcome Trust Case Control Consortium 2) with two independent case–control datasets of individuals of European descent and all cases had been diagnosed as having psoriasis vulgaris^16^. Participants of CD GWAS were recruited from the UK, Finland, the Netherlands and Italy, and the diagnosis of CD was based on ﻿standard clinical, serological and histopathological criteria, including small intestinal biopsy^17^. The detailed methods for sample collection, genotyping and quality control can be found in the original studies^16,17^.

### Instrument identification

Eligible IVs for MR should meet the following 3 assumptions^10^. Assumption 1 is that IVs should be related to exposure^10^. To ensure our IVs are strong, we restricted the set of single nucleotide polymorphisms (SNPs) to associations with a p-value smaller than 5E−08 as potential instruments; moreover, we removed weak instruments with F-statistics < 10^10^. Assumption 2 is that IVs should be unrelated to any other risk factor/confounding that may affect the outcome variable, and assumption 3 is that IVs should not have a direct association with outcome, both of which are also known as horizontal pleiotropy, which could be calculated as MR Egger intercept in the post-MR analysis^10^.

### Bidirectional two-sample MR analysis

Once the IVs were selected, independent SNPs were set at a threshold of linkage disequilibrium (LD) at r^2^ = 0.001 within the window of 10 megabase pairs to avoid double counting and biased causal effect estimates. Next, the IVs were extracted from the outcome trait and were harmonized in both exposure and outcome GWAS. In this step, palindromic SNPs with intermediate allele frequency were removed. Moreover, if a particular requested SNP is not present in the outcome GWAS, then an SNP (proxy) that is in LD with the requested SNP (target) will be searched, which was defined using 1000 genomes European sample data (r^2^ ≥ 0.8). Once the exposure and outcome data are harmonized, MR can be performed. The inverse variance weighted (IVW) method was performed as the main analysis, which is the most efficient analysis method with valid IVs because it accounts for heterogeneity in the variant-specific causal estimates^18^. Moreover, additional sensitivity analyses including the simple mode, weighted mode, weighted median, and MR-Egger regression methods, were further conducted to assess the robustness of the findings^18^. And the leave-one-out analysis was conducted within the IVW method to assess the influence of individual variants on the observed association. Moreover, to ensure the causation was not distorted by the presence of reverse causation, the Steiger filtering test was applied, and p < 0.05 indicated that the effect direction is from exposure to an outcome^19^. Furthermore, post-MR analyses including Cochran's Q test for heterogeneity, MR Egger Intercept test for horizontal pleiotropy and MRPRESSO test for the outlier were also performed^20^. Lastly, we computed the proportion of variance in the phenotype explained by IVs and calculated the statistical power for the MR study with a two-sided type-I error rate α = 0.05^21^. The main statistical analyses were conducted using TwoSampleMR (v.0.5.5) in R package (V.4.1.2)^22^. The study was performed according to the STROBE MR guideline^23^.

## Results

**Figure 2:**
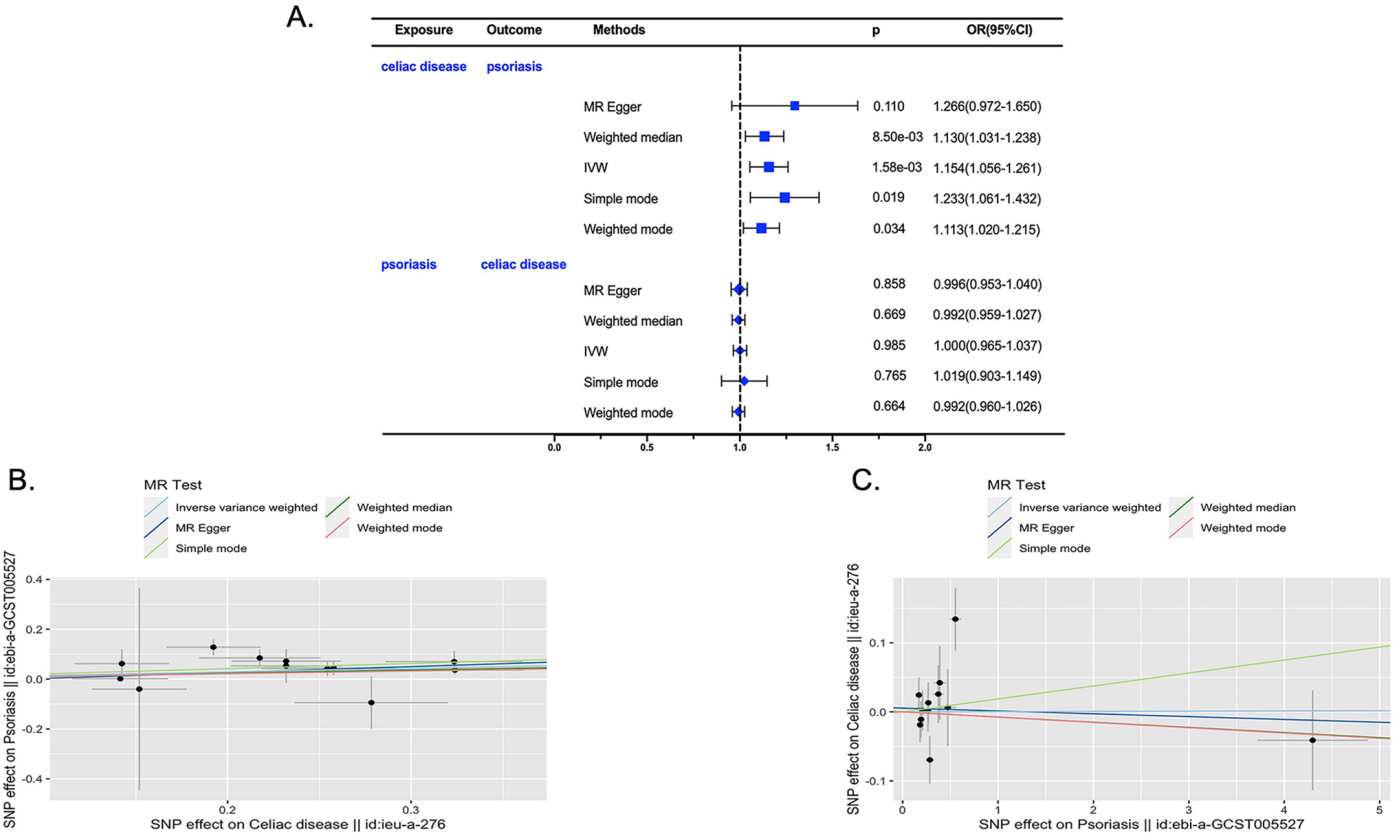
Genetic association between psoriasis and CD using bidirectional 2-sample MR analysis. (**A**) Forest plot summarizing the bi-directional MR results: the effect of CD on psoriasis (upper panel); the effect of psoriasis on CD (lower panel); *OR* odds ratio; *CI* confidential interval. *IVW* inverse variance weighted. (**B**) Scatter plot of the effect of celiac disease on psoriasis; (**C**) Scatter plot of the effect of psoriasis on Celiac disease.

On the one hand, we studied the causal effect of CD on psoriasis. Twelve IVs were available after clumping (Supplementary Table S1). We found that each standard deviation of a genetically determined increase in CD was associated with approximately 20% increased risk of psoriasis (p = 1.58e−03, OR [95% CI] 1.232 [1.061–1.432]). And the results were further supported by weighted median (p = 8.50e−03, OR [95% CI] 1.130 [1.031–1.237]), simple mode (p = 0.019, OR [95% CI] 1.232 [1.061–1.432]) and weighted mode (p = 0.020, OR [95% CI] 1.113[1.020–1.215]). And the MR-Egger analysis also showed the same trend although did not reach statistical significance (p = 0.110, OR [95% CI] 1.266[0.972–1.650]) (Fig. 2A,B and Supplementary Table S3).

On the other hand, we studied the causal effect of psoriasis on CD. Twelve IVs were included (Supplementary Table S2). We found that genetically determined psoriasis was not associated with the risk for CD (IVW: p = 0.985, OR [95% CI] 1.000[0.965–1.037]), and the results were further supported by sensitivity analyses (Fig. 2A,C and Supplementary Table S3).

Next, we performed extra analysis to confirm the causal association between psoriasis and CD. The Steiger-filtering test indicated that the direction of causality was correct (p < 0.05). And the intercepts of MR-Egger were both not significantly deviated from zero, suggesting no apparent horizontal pleiotropy. The Cochran’s Q test did not detect any heterogeneity among the IVs, and the MR-PRESSO analysis did not detect potential instrumental outliers at the nominal significance level of 0.05. The leave-one-out results suggest that the causal effect was not driven by a single IV (Supplementary Fig. S1). Moreover, in the statistical power test, the power of assessing the causality of CD on psoriasis was 0.83, while the power of assessing the causality of psoriasis on CD was only 0.05(Table 1).

**Table 1:** Horizontal pleiotropy, heterogeneity, and outlier test for the bidirectional 2-sample MR analysis.

Exposure	Outcome	Horizontal pleiotropy			Heterogeneity						Global test pvalue in MR-PRESSO	Steiger test		Power
		egger_intercept	se	pval	MR-Egger			IVW				steiger_pval	Correct causal direction	
					Q	Q_df	Q_pval	Q	Q_df	Q_pval				
Celiac disease	Psoriasis	− 0.021	0.028	0.479	18.936	10	0.041	19.958	11	0.460	0.080	2.39e−73	True	0.83
psoriasis	Celiac disease	0.005	0.014	0.706	15.753	10	0.107	15.989	11	0.141	0.391	9.04e−34	True	0.06

## Discussion

It has been widely studied that patients with psoriasis and patients with CD were both more likely to develop other autoimmune diseases including type 1 diabetes, autoimmune thyroiditis, rheumatoid arthritis, et al^24,25^. Moreover, a meta-analysis also found a higher prevalence of psoriasis in patients with CD, as well as a higher prevalence of CD in patients with psoriasis^8^. However, observational studies were unable to avoid confounding factors and reverse causality. In the current study, we studied the bidirectional genetic relationship between these two autoimmune diseases with an unbiased MR design. And we provided novel genetic evidence that CD was associated with an increased risk of psoriasis, while psoriasis was not associated with the risk of CD.

CD and psoriasis are both immune-mediated diseases, more specifically, they are both T-cell mediated disorders^26,27^, and the immunogenic mechanisms might explain the causal link between CD and psoriasis. CD is a T-cell mediated immune disease in which gliadin-derived peptides activate lamina propria effector CD4 + T cells^28^. This activation could lead to the release of cytokines (including interferon-gamma, transforming growth factor-β, interleukin 2, and interleukin 10), as well as the activation of downstream effector T cells (including T-helper 1 cells, T-helper 17 cells, and regulatory T cells)^28^. And patients with CD were having altered intestinal barrier dysfunction, therefore, the proinflammatory cytokines and activated T cells could enter the blood with a subsequent increase in the dermis and epidermis, and ultimately lead to the development of psoriasis^29^.

Another mechanism of CD increasing the risk of psoriasis could be attributable to the gluten-free diet in patients with CD. On the one hand, gluten-free diet could lead to deficiencies in many kinds of vitamins (such as Vitamin D and Vitamin B12) and nutrients, such as iron, calcium and folate^30^, which could contribute to the development of psoriasis. For example, low vitamin D levels could involve in the pathogenesis of psoriasis through pathways including dysregulating keratinocytes proliferation, differentiation, and apoptosis, dysregulating of the cutaneous immune system (inhibition of T cell proliferation, Tregs induction), upregulating pro-inflammatory cytokines, inhibiting antimicrobial peptides expression, and destroying barrier integrity and permeability^31^. And Vitamin B12 could scavenge nitric oxides and reactive oxidative stress (ROS), while low levels of Vitamin B12 could exacerbate inflammatory oxidative stress^32^. On the other hand, gluten-free diet led to a higher content of both saturated and hydrogenated fatty acids^30^. Saturated fatty acids could sensitize myeloid cells to an increased inflammatory response in answer to proinflammatory stimuli, which in turn augments the activation of keratinocytes and exacerbates psoriasis^33^. In general, gluten-free diet plays an important role in the causality of CD on psoriasis.

There are some limitations that should be acknowledged in the current study. First, it is that completely excluding pleiotropy (assumption 2) is challenging for all MR analyses, because some unknown confound factor could influence both exposure and outcome; however, we applied MR-Egger intercept and MR-PRESSO to verify the lack of horizontal pleiotropy in the current study. Moreover, the statistical power of assessing the causality of psoriasis on CD was relatively low (0.05), which indicated that larger-sample-size GWAS of psoriasis would be necessary. Briefly, with a desired statistical power at 0.8 and with a presupposed OR at 1.2, a sample size of 14,210 cases and 33,156 cases would be required. Last but not least, the GWAS datasets used in the current study were both derived from the European population, more studies from other ethnic groups are needed to confirm the results.

## Conclusion

Our study provided novel genetic evidence that patients with CD were at an increased risk of developing psoriasis, while psoriasis was not associated with the risk for CD. Clinicians should be aware of the associations and pay attention to skin manifestations in patients with CD.

## Supplementary Information

Supplementary Figure 1.

Supplementary Tables.

## Data Availability

The datasets used and/or analyzed during the current study are available from the corresponding author upon reasonable request.

## References

[1] Armstrong AW, Read C (2020). Pathophysiology, clinical presentation, and treatment of psoriasis: A review. JAMA J. Am. Med. Assoc..

[2] Boehncke WH, Schön MP (2015). Psoriasis. Lancet.

[3] Progress M, Green PHR, Cellier C (2007). (Celiac disease) Celiac disease. Management.

[4] De Bastiani R (2015). Association between coeliac disease and psoriasis: Italian primary care multicentre study. Dermatology.

[5] Birkenfeld S, Dreiher J, Weitzman D, Cohen AD (2009). Coeliac disease associated with psoriasis. Br. J. Dermatol..

[6] Montesu MA (2011). Association between psoriasis and coeliac disease? a case-control study. Acta Derm. Venereol..

[7] Ojetti V (2003). High prevalence of celiac disease in psoriasis. Am. J. Gastroenterol..

[8] Acharya P, Mathur M (2020). Association between psoriasis and celiac disease: A systematic review and meta-analysis. J. Am. Acad. Dermatol..

[9] Lee YH (2020). Overview of Mendelian Randomization Analysis. J. Rheum. Dis..

[10] Lee K, Lim C-Y (2019). Mendelian randomization analysis in observational epidemiology. J. Lipid Atheroscler..

[11] Bowden J, Holmes MV (2019). Meta-analysis and Mendelian randomization: A review. Res. Synth. Methods.

[12] Davies NM, Holmes MV, Davey Smith G (2018). Reading Mendelian randomisation studies: A guide, glossary, and checklist for clinicians. BMJ.

[13] Patrick MT (2021). Causal relationship and shared genetic loci between psoriasis and type 2 diabetes through trans-disease meta-analysis. J. Invest. Dermatol..

[14] Wu D (2021). The causal effect of interleukin-17 on the risk of psoriatic arthritis: A Mendelian randomization study. Rheumatology.

[15] Budu-Aggrey A (2019). Evidence of a causal relationship between body mass index and psoriasis: A mendelian randomization study. PLoS Med..

[16] Tsoi LC (2012). Identification of 15 new psoriasis susceptibility loci highlights the role of innate immunity. Nat. Genet..

[17] Dubois PCA (2010). Multiple common variants for celiac disease influencing immune gene expression. Nat. Genet..

[18] Burgess S (2019). Guidelines for performing Mendelian randomization investigations. Wellcome Open Res..

[19] Hemani G, Tilling K, Smith GD (2017). Orienting the causal relationship between imprecisely measured traits using genetic instruments. PLoS Genet..

[20] Verbanck M, Chen C-Y, Neale B, Do R (2018). Detection of widespread horizontal pleiotropy in causal relationships inferred from Mendelian randomization between complex traits and diseases. Nat. Genet..

[21] Brion MJA, Shakhbazov K, Visscher PM (2013). Calculating statistical power in Mendelian randomization studies. Int. J. Epidemiol..

[22] Hemani G (2018). The MR-base platform supports systematic causal inference across the human phenome. Elife.

[23] Skrivankova VW (2021). Strengthening the reporting of observational studies in epidemiology using mendelian randomization: The STROBE-MR statement. JAMA.

[24] Woo YR, Park CJ, Kang H, Kim JE (2020). The risk of systemic diseases in those with psoriasis and psoriatic arthritis: From mechanisms to clinic. Int. J. Mol. Sci..

[25] Lauret E, Rodrigo L (2013). Celiac disease and autoimmune-associated conditions. Biomed Res. Int..

[26] Jabri B, Sollid LM (2017). T cells in celiac disease. J. Immunol..

[27] Cai Y, Fleming C, Yan J (2012). New insights of T cells in the pathogenesis of psoriasis. Cell. Mol. Immunol..

[28] Mazzarella G (2015). Effector and suppressor T cells in celiac disease. World J. Gastroenterol..

[29] Krueger JG, Bowcock A (2005). Psoriasis pathophysiology: Current concepts of pathogenesis. Ann. Rheum. Dis..

[30] Vici G, Belli L, Biondi M, Polzonetti V (2016). Gluten free diet and nutrient deficiencies: A review. Clin. Nutr..

[31] Barrea L (2017). Vitamin D and its role in psoriasis: An overview of the dermatologist and nutritionist. Rev. Endocr. Metab. Disord..

[32] Kanda N, Hoashi T, Saeki H (2020). Nutrition and psoriasis. Int. J. Mol. Sci..

[33] Herbert D (2018). High-fat diet exacerbates early psoriatic skin inflammation independent of obesity: Saturated fatty acids as key players. J. Invest. Dermatol..

