# Supplementary material for: Mendelian randomization study of the genetic interaction between psoriasis and celiac disease
Source: Sci Rep. 2022 Dec 13;12:21508. doi: 10.1038/s41598-022-25217-y (PMC9747804; doi:10.1038/s41598-022-25217-y)
Supplement: Supplementary file 1 — Supplementary Figure 1. [file 41598_2022_25217_MOESM1_ESM.pdf]

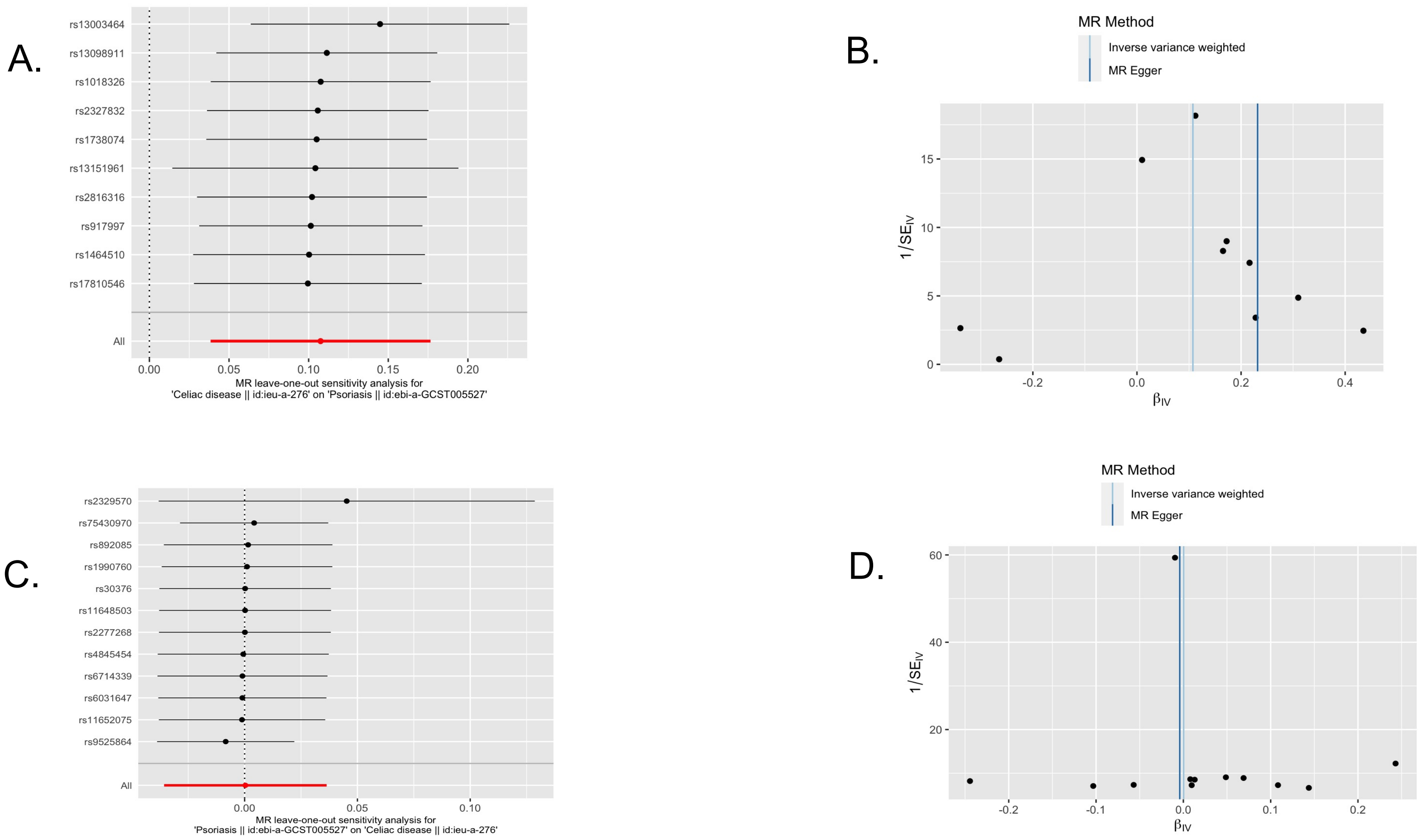

Supplementary Figure 1: Leave-out-one analysis and funnel plots. A. The leave-one-out results suggested that celiac disease's causal effect on psoriasis was not driven by a single IV. B. Funnel plot for celiac disease's effect on psoriasis. C. The leave-one-out results suggested that psoriasis's causal effect on the celiac disease was not driven by a single IV. D. Funnel plot for psoriasis's effect on celiac disease.
